# Modelling rate of penetration in drilling operations using RBF, MLP, LSSVM, and DT models

**DOI:** 10.1038/s41598-022-14710-z

**Published:** 2022-07-08

**Authors:** Mohsen Riazi, Hossein Mehrjoo, Reza Nakhaei, Hossein Jalalifar, Mohammadhadi Shateri, Masoud Riazi, Mehdi Ostadhassan, Abdolhossein Hemmati-Sarapardeh

**Affiliations:** 1 Department of Petroleum Engineering, Shahid Bahonar University of Kerman, Kerman, Iran; 2 Enhanced Oil Recovery (EOR) Research Center, IOR/EOR Research Institute, Shiraz University, Shiraz, Iran; 3 Département de génie des systèmes, École de technologie supérieure, Montreal, QC Canada; 4 Key Laboratory of Continental Shale Hydrocarbon Accumulation and Efficient Development, Ministry of Education, Northeast Petroleum University, Daqing, 163318 China; 5 Institute of Geosciences, Marine and Land Geomechanics and Geotectonics, Christian-Albrechts-Universität, 24118 Kiel, Germany; 6 Department of Geology, Ferdowsi University of Mashhad, Mashhad, Iran

**Keywords:** Solid Earth sciences, Energy science and technology, Engineering

## Abstract

One of the most important problems that the drilling industry faces is drilling cost. Many factors affect the cost of drilling. Increasing drilling time has a significant role in increasing drilling costs. One of the solutions to reduce drilling time is to optimize the drilling rate. Drilling wells at the optimum time will reduce the time and thus reduce the cost of drilling. The drilling rate depends on different factors, some of which are controllable and some are uncontrollable. In this study, several smart models and a correlation were proposed to predict the rate of penetration (ROP) which is very important for planning a drilling operation. 5040 real data points from a field in the South of Iran have been used. The ROP was modelled using Radial Basis Function, Decision Tree (DT), Least Square Vector Machine (LSSVM), and Multilayer Perceptron (MLP). Bayesian Regularization Algorithm (BRA), Scaled Conjugate Gradient Algorithm and Levenberg–Marquardt Algorithm were employed to train MLP and Gradient Boosting (GB) was used for DT. To evaluate the accuracy of the developed models, both graphical and statistical techniques were used. The results showed that DT-GB model with an R^2^ of 0.977, has the best performance, followed by LSSVM and MLP-BRA with R^2^ of 0.971 and 0.969, respectively. Aside from that, the proposed empirical correlation has an acceptable accuracy in spite of simplicity. Moreover, sensitivity analysis illustrated that depth and pump pressure have the highest effects on ROP. In addition, the leverage approach approved that the developed DT-GB model is valid statistically and about 1% of the data are suspected or out of the applicability domain of the model.

## Introduction

One of the most important issues facing the oil industry, especially the drilling industry, is the costs of drilling, and has attracted much attention in recent decades. Many factors can affect the cost of drilling, the most important of which is the drilling time of the well, which can increase drilling costs several times. Therefore, reducing drilling time is one of the most significant purposes of drilling engineers^1–3^. In other words, one of the major aims of drilling optimization is to lessen the total time^4^. For this purpose, two ways have been proposed: choosing optimum drilling variables (e.g. picking a suitable drilling fluid type and drill-bit) and instantaneous analysis so as to optimize operational parameters such as rotary speed and weight on bit while drilling^4^.

**Figure 1:**
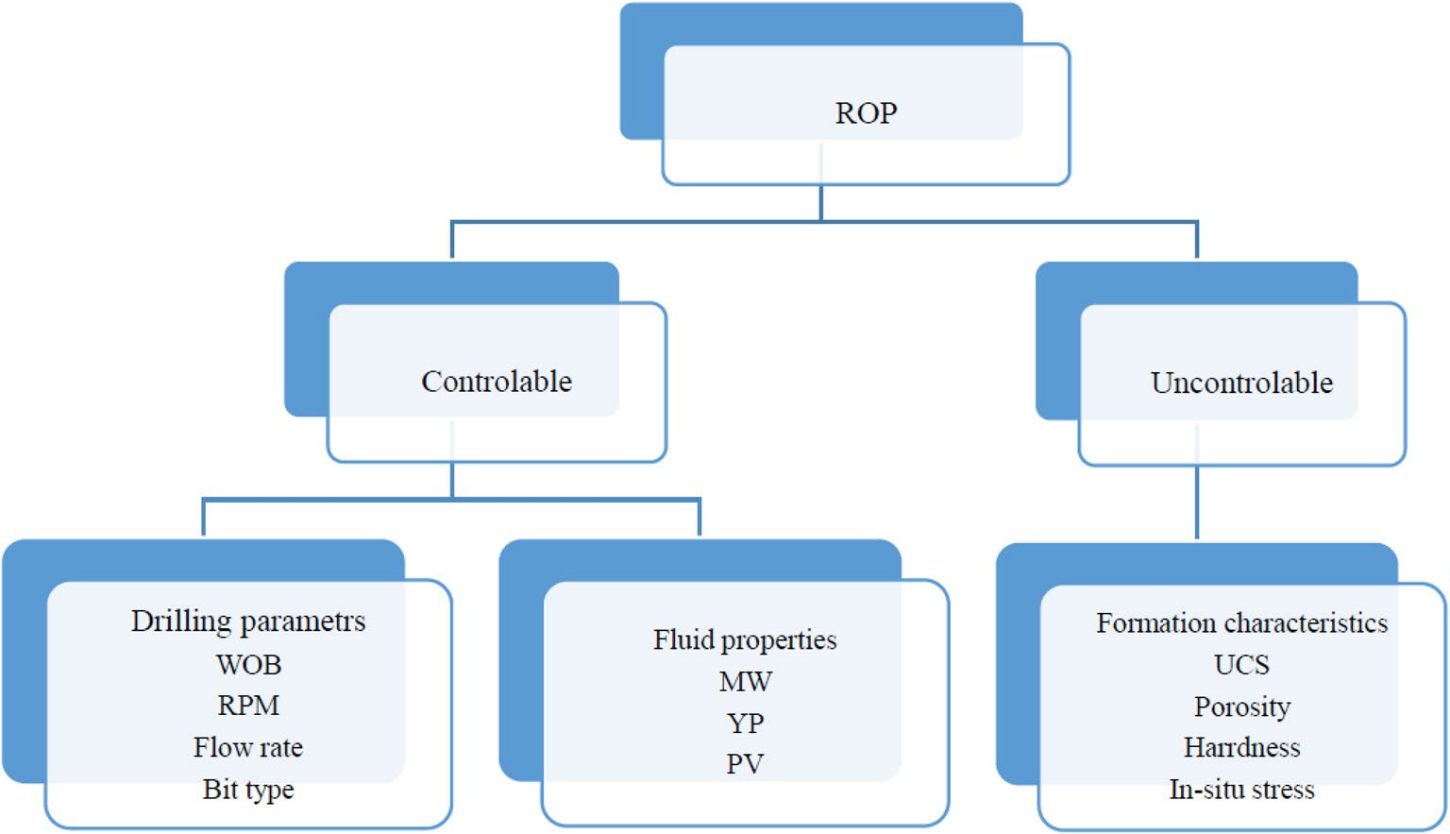
The main factors affecting ROP.

The major factor affecting drilling time is the rate of penetration (ROP)^5^. Hence, the precision of ROP model is critical^6^. Many parameters affect the drilling rate, including drilling mud properties, formation characteristics, rotary speed, and bit characteristics^2,7^. The main parameters that affect ROP are presented in Fig. 1. Some of these parameters are uncontrollable, such as formation characteristics, and others are controllable, such as the properties of drilling mud. Studying the effect of different parameters individually on ROP can easily be investigated, such as rock strength, revolutions per minute (RPM), and weight on bit (WOB)^8^. Increasing uniaxial compressive strength of formation rock causes hardening and thus decreases penetration rate^8,9^. The drilling parameters are also controllable factors for changing drilling rate. The type of bit and its genus^10^, and the fit of bit and formation are effective in increasing drilling rate. Although increasing RPM^11^ increases drilling rate in soft formations, it is not directly visible in hard formations and low rotational speeds can result in better drilling rates. The flow rate and characteristics of drilling mud, such as plastic viscosity (PV), mud weight (MW), and yield point (YP) determine the ability of the mud to transfer drilling cuttings to the surface. Better cutting transportation to the surface prevents the accumulation of cuttings and regrinding, and increases drilling rate. The WOB determines the degree of contact and penetration of bit into the formation which depends upon the type of rock, and can increase the drilling rate, but the WOB has a direct relation to the drilling rate to a certain extent, and then has no great impact on drilling rate^12,13^. Many models have been proposed to predict ROP, but they are problematic as they are obtained either in the lab or by using incomplete field data^2,14^. In other word, effects of the drilling variables on the ROP has not yet been understood completely^15^. So far, different methods have been proposed to optimize the drilling rate, but due to the fact that a large number of parameters influence the drilling rate, development of an efficient and comprehensive model is very difficult. On the other hand, the complex relationship between these parameters has led to a lack of a comprehensive model^2,14^.

Normally, two main approaches are used to predict ROP, including traditional models and machine learning (ML) models.

Some famous traditional correlations are as follows:

Maurer^16^ developed Eq. (1) for rolling cutter bits:

1 $$ROP = \frac{K}{{S^{2} }}\left( {\frac{W}{{d_{b} }} - \frac{{W_{o} }}{{d_{b} }}} \right)^{2} N.$$

In the above equation, S and K are the compressive rock strength and constant of proportionality, respectively. W_o_ and W are the threshold bit weight and bit weight, respectively. d_b_ shows diameter of drill-bit and N denotes the rotary speed.

Another traditional model for ROP was introduced by Bingham^17^:

2 $$ROP = K\left( {\frac{W}{{d_{bit} }}} \right)^{{a_{5} }} N.$$

where R, W, $d_{bit}$, and N refer to ROP (ft/hr), weight on bit (klbs), bit diameter (in), and rotary speed (rot/min), respectively. K and $a_{5}$. are Bingham coefficients, and have different values for various formations^18^.

One of the most important ROP models was developed by Bourgoyne and Young^19^. This model is extensively employed in the industry^20^. Equation (3) was proposed by Bourgoyne and Young^19^. Eight parameters were involved in Bourgoyne and Young model^19^ (BYM).

3 $$\frac{dD}{{dt}} = {\text{exp}}\left( {a_{1} + \mathop \sum \limits_{j = 2}^{8} a_{j} x_{j} } \right)$$

where D shows the well depth, the coefficient a_1_to a_8_ are associated with the formation strength parameter, formation compaction, pore pressure, differential pressure, weight on bit exponent, rotary drilling, drill-bit tooth wear, and bit hydraulic jet impact, respectively, and t denotes the time. Afterwards, Bourgoyne et al.^18^ suggested an adaptation to their original ROP model:

4 $$ROP = \left( {f_{1} } \right)*\left( {f_{2} } \right)* \ldots \left( {f_{8} } \right)$$

In the above equation, the functions f_1_ to f_8_ involves the empirical coefficients a_1_–a_8_. As stated by Soares and Gray^6^, the main difference between Eqs. (3) and (4) is in the last function. Equation (3) uses Eckel's hydraulics Reynolds number, however in Eq. (4) a power law function of the hydraulic jet impact force was used. Although the BYM equations denote all important features of drilling, some parameters which are necessary in the model are not simply measured in real-time (e.g. drill bit wear, differential pressure)^6^.

A general drag bit model was introduced by Hareland and Rampersad^21^:

5 $$ROP = \frac{{14.14*N_{c} NA_{v} }}{{d_{b} }}$$

where, N_c_ and A_v_ show the number of cutters and the area of rock compressed ahead of a cutter, which supposes a different formulation based on the drill-bit type, respectively. More details can be found in Soares et al. work^22^.

Finding the definite connection among the drilling parameters is not well realized and is very difficult^15^. Hence, some researches^23–25^ have been made to better comprehend the connection among the drilling parameters and how they affect the ROP. For instance, Motahhari et al.^23^ suggested an ROP model for polycrystalline diamond compact (PDC) bit:

6 $$ROP = W_{f} \frac{{GN^{y} N^{\alpha } }}{{d_{b} S}}$$

In this equation, S shows confined rock strength. $\alpha$ and y represent the coefficient of ROP model and W_f_ denotes wear. G presents coefficient related to bit geometry and bit-rock interactions. Deng et al.^24^ suggested a theoretical model for ROP. This model was developed for roller cone bit and it was validated with results that were achieved from experimental data. In this model, the rock dynamic compressive strength was used in reverse static compressive strength, which improved the accuracy of the theoretical model. Eq. (7) developed by Al-AbdulJabbar et al.^25^ and it is based on regression analysis:

7 $$ROP = 16.96\frac{{W^{a} *N*T*SSP*Q}}{{PV*\rho *d_{b}^{2} *UCS^{b} }}$$

where 16.96 is used to converted units, ${\uprho }$ shows the mud density, T denotes the torque, SSP represents the standpipe pressure, Q shows the flow rate, PV presents the plastic viscosity, UCS denotes the uniaxial compressive strength. Nonlinear regression was used to calculate the coefficients (a and b).

Equation (8) proposed by Warren:

8 $$ROP = { }\left( {\frac{{aS^{2} d_{b}^{3} }}{{NW^{2} }} + \frac{c}{{Nd_{b} }}} \right)^{ - 1}$$

where S shows rock strength, and ${\text{a}}$ and ${\text{c}}$ denote constant^8^.

Effects of other factors, such as hold down of chip^26,27^, bit wear^28^, and cutting geometry ^29,30^ was considered by many researchers. Eckel^31^ expressed that mud properties have no direct effect on ROP, while Paiaman et al.^32^ showed that growing the plastic viscosity and mud weight can decrease the rate of penetration. Moraveji et al.^33^ developed a model and illustrated that the gel strength, WOB and YP/PV ratio have remarkable effect on ROP.

Soares et al.^22^ showed limitations of traditional ROP mods such as model introduced by Bourgoyne et al.^19^. ML methods are interesting methods to predict ROP. Priority of machine learning techniques than traditional model was proved by several researchers^8,34–36^. The first work about prediction of ROP by ML was conducted by Bilgesu et al.^37^. The ability of the neural networks to find a complex relationship between data has led to this approach being taken to predict drilling rates. Nowadays, artificial neural networks (ANNs) are widely used in oil industry. We briefly mention few of them in the following part. Alarfaj et al.^38^ predicted ROP using ANNs and compared several models. They concluded that the extreme learning machine (ELM) gives the accurate results. They did not consider the effect of flow rate, RPM, MW and bit diameter in their models. Ansari et al.^39^ used error analysis of multivariate regression to select the best parameters to predict ROP and then used support vector regression (SVR) techniques to model ROP. Finally, committee support vector regression (CSVR) based on imperialist competitive algorithm (ICA) was employed to predict ROP. Their results showed that CSVR-ICA model can improve the result of SVR^39^. Hegde et al.^36^ conducted evaluation of two different approaches, physics-based and data-driven modeling approaches, for prediction of ROP. Their results showed that the data-driven model had better prediction than traditional models^36^. Soares and Gray^6^ studied real-time predictive capabilities of ML and analytical ROP models. Their results showed than ML models decrease the error much better than analytical ones. In addition, among analytical models, the best performance belonged to BYM^6^. Ashrafi et al.^40^ employed hybrid artificial intelligence models to predict ROP. Based on their results, particle swarm optimization-multi-layer perception (PSO-MLP) gained the best performance^40^. Usage of ANN for ROP prediction during drilling operation was also evaluated by Diaz et al.^41^. Gan et al.^42^ suggested a new hybrid modeling model to estimate ROP. Excellent prediction performance of their proposed model was shown in this study^42^. Mehrad et al.^43^ used mud logging and geomechanical parameters to predict ROP by hybrid algorithm. Least-square support-vector machines-cuckoo optimization algorithm (LSSVM-COA) had the best performance among used models. The difference of errors in training and testing data of the developed model by LSSVM-COA was small^43^.

This study is conducted to develop an empirical correlation and some smart models including least square vector machine (LSSVM), multilayer perceptron (MLP), Decision Tree (DT), and Radial Basis Function (RBF), for ROP based on a large data bank (more than 5000 data points) obtained from drilling in South fields of Iran. Gradient boosting (GB) is used for DT optimization and Bayesian Regularization Algorithm (BRA), Scaled Conjugate Gradient Algorithm (SCGA) and Levenberg-Marquardet Algorithm (LMA) are used to train MLP modes. What distinguishes this study is to consider more effective parameters in developing the models. These parameters include depth (D), weight on bit (WOB), pit total (PT), pump pressure (PP), hookload (H), surface torque (ST), rotary speed (RS), flow in (Fi), flow out (Fo), and wellhead pressure (Wp). The accuracy and validity of the proposed models are evaluated by statistical and graphical techniques. In addition, the Leverage approach is employed to check the validity of the experimental data and applicability domain of the proposed models.

## Modelling

### Generalized reduced gradient (GRG)

For developing an empirical correlation for ROP, we proposed a structure for the correlation and used Generalized reduced gradient (GRG) to optimize the coefficient of the correlation. The optimum structure was obtained by a trial-and-error procedure. GRG is known as one of the techniques for solving multivariable problems. This method is used to solve both nonlinear and linear problems^44^. In this method, variables are regulated to continue the active restrictions being satisfied once the process shifts from one point to another. Linear guess to the gradient at a specific point y is developed by GRG. Both the objective gradient and restriction are solved alongside. The objective gradient function can be denoted in the form of the gradients of restrictions. Later, the search can move in a realistic way and the search area's size is reduced. For an objective function f(y) subjected to h(y)^45^:

9 $$Minimize: \, f\left( y \right) = y$$

10 $$Subjected \, to: h_{k} \left( y \right) = 0$$

GRG can be stated as follows^45^:

11 $$\frac{df}{{dy_{k} }} = \nabla y_{k}^{t} f - \nabla y_{i}^{t} f\left( {\frac{\partial h}{{\partial y_{i} }}} \right)^{ - 1} \left( {\frac{\partial h}{{\partial y_{k} }}} \right)$$

One of the vital conditions for f(y) to be minimized is that df(y) = 0. Interested readers can achieve more details in the literature^46–49^.

### Decision Tree (DT)

**Figure 2:**
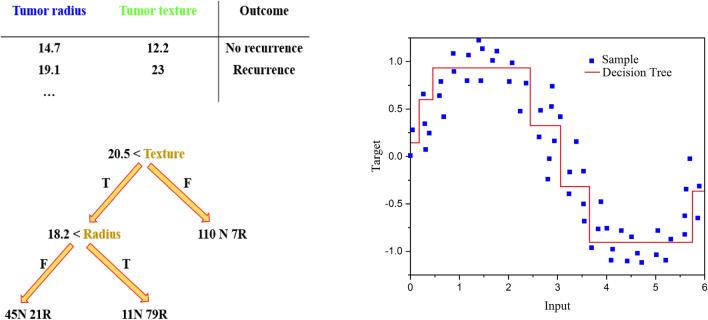
The schematic diagram of DT.

DT is known as a non-parametric supervised learning method that can be applied for both classification and regression problems. Morgan and Sonquist^50^ introduced Automatic Interaction Detection (AID), known as the first decision tree. Messenger and Mandell^51^ introduced THeta Automatic Interaction Detection (THAID), the first classification tree algorithm. THAID is a hierarchical flow chart involving branches, root nodes, internal nodes, and leaf nodes. A top node that does not have any income branch is called root node. The root node presents the entire sample space. Nodes contain one incoming branch and more outgoing edges are the internal or test nodes. Leaves or terminal nodes are nodes that show the final results. Pruning, stopping, and splitting are three main procedures for making a decision tree^52^. Separating the data into a number of subsets, based on testing the most noted attribute that is valid also for the training instances is accomplished in the splitting step. Various criteria such as Gini index, information gain, gain ratio, information gain, classification error, and towing could be considered for standard deviation reduction, variance reduction, and classification tree^53^. Figure 2 shows an instance of a decision tree that is used for both regression and classification. Data splitting is started from the root node and develops to the internal node until reaching the stopping criteria or satisfaction of the predefined homogeneity. Representing the stopping criteria can result in a lessening of the problem complexity. This approach results in avoiding overfitting. Splitting would cause a complex tree once stopping criteria are not applied. Although the training data would be fitted well, it does not occur for the test data. Usage of represented stopping criteria would be restricted to tuning the model for the best value. In order to avoid overfitting, if stopping methods do not work properly, pruning technique is applied. In pruning technique, a complete tree is made. Afterward, it is pruned to small trees which are generated by the removal of some nodes that contain less information gain or validation data.

### Radial basis function (RBF) neural network

RBF and MLP are the most widely used artificial neural network (ANN) models. With these differences that the RBF model has a simpler design and its structure is fixed and consists of only three layers. It should also be noted that the categorization methods are unalike between the MLP and RBF. The data values in this method are gained based on the space of the points from the points called the center. Centers are chosen in three different ways: (a) supervised, (b) unsupervised (c) fixed. In each neuron, a transport function acts, thus, we have for f(z_i_) = output:

12 $$f\left( {z_{i} } \right) = { }\emptyset \left( {z_{i} } \right) \times w^{t} + b$$

where terms $\emptyset \left( {z_{i} } \right)$, $w^{t}$ and $b$ refer to transport function, transposed matrix of weights, and bias vector, respectively.

Equation (13) shows Gaussian function and generally it is the transport function in RBF models:

13 $$\emptyset \left( r \right) = \exp \left( {0.5 \times \left( {\frac{r}{\sigma }} \right)^{2} } \right)\;{\text{for}}\;\sigma {\text{ > }}0$$

Other common radial functions are:

14 $$\emptyset \left( r \right) = \sqrt {1 + \left( {\frac{r}{\sigma }} \right)^{2} }$$

15 $$\emptyset \left( r \right) = \frac{1}{{\sqrt {1 + \left( {\frac{r}{\sigma }} \right)^{2} } }}$$

16 $$\emptyset \left( r \right) = 1 + \left( {\frac{r}{\sigma }} \right)^{2}$$

17 $$\emptyset \left( r \right) = { }r^{2} \ln \left( r \right)$$

The distance of point $z_{i}$. from center $c_{k}$. is shown as, $\left\| {z_{k} - c_{i} } \right\|$, thus, we have:

18 $$\varphi _{{ki}} \left( z \right) = \exp \left( {0.5 \times \frac{{\left\| {z_{k} - c_{i} } \right\|}}{{\sigma ^{2} }}} \right),\;i = 1, \ldots ,N\;{\text{and}}\;k = 1, \ldots ,M$$

The number of inputs and kernels, centers, and Gaussian transport function is symbolized by, N, M, $c_{k}$. and $\varphi_{ik} \left( z \right)$, respectively.

According to the above statements, outputs are obtained by^54–57^:

19 $$output = f_{k} \left( {z_{i} } \right) = w_{0} + \sum\limits_{{i = 1}}^{N} {\varphi _{{ki}} } \times w_{i} \times \left( {\left\| {z_{k} - c_{i} } \right\|} \right),i = 1, \ldots ,N\;{\text{and}}\;k = 1, \ldots ,M$$

**Figure 3:**
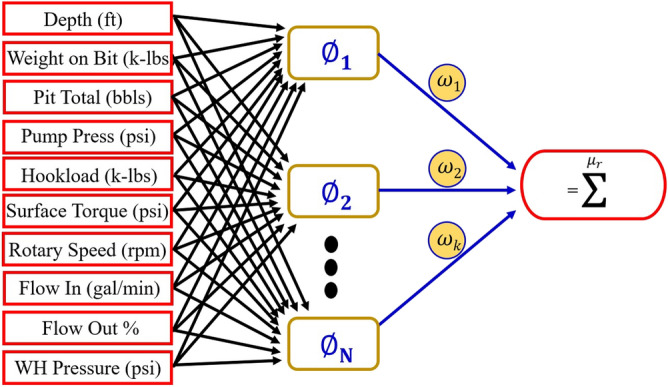
The schematic diagram of RBF.

**Figure 4:**
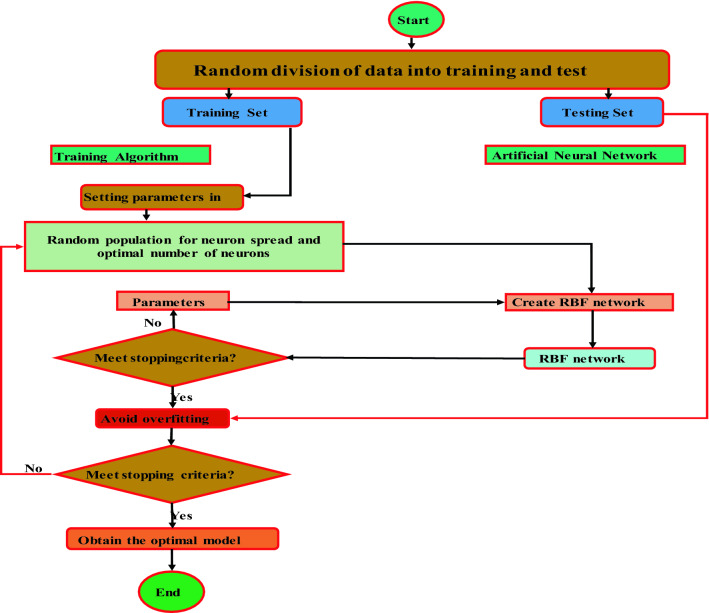
Flowchart for the suggested RBF model^72^.

The schematic of RBF model and flowchart for the proposed RBF model illustrated in Figs. 3 and 4, respectively. The spread coefficient and the maximum number of neurons in RBF are 2 and 100, respectively. In addition, Gaussian function was used as a transfer function in the present study for RBF model.

### Multilayer perceptron (MLP)

MLP is a feed-forward ANN that can have several layers. A simple MLP model consists of at least three layers. In this case, a hidden layer connects input and output layer. The layers are composed of neurons, except for the input layer, the neurons of the other layers contain a nonlinear activation function. The number of layers and neurons in each layer could be determined by considering the number of input data and complexity of the problem. Learning this network is performed using a supervised back-propagation algorithm. Weights and bias are the parameters of each neuron. Several functions can be used as a transfer function in hidden and output neurons. Some of these functions are presented below:

20 $$Binary \, step: \, f\left( z \right) = \left\{ {\begin{array}{*{20}l} z \hfill & {for} \hfill & {z < 0} \hfill \\ { - z} \hfill & {for} \hfill & {z \ge 0} \hfill \\ \end{array} } \right.$$

21 $${\text{Tansig}}: \, f\left( z \right) = { }\frac{{e^{z} - e^{ - z} }}{{e^{z} + e^{ - z} }} = \frac{2}{{1 + e^{ - 2z} }} - 1$$

22 $${\text{Logsig}}: \, f\left( z \right) = \frac{1}{{1 + e^{ - z} }}$$

23 $$ArcTan:f\left( z \right) = { }tan^{ - 1} \left( z \right){ }$$

24 $$inusid:f\left( z \right) = {\text{sin}}\left( z \right)$$

25 $$Purelin:{ }f{ }\left( z \right){ } = { }z$$

**Figure 5:**
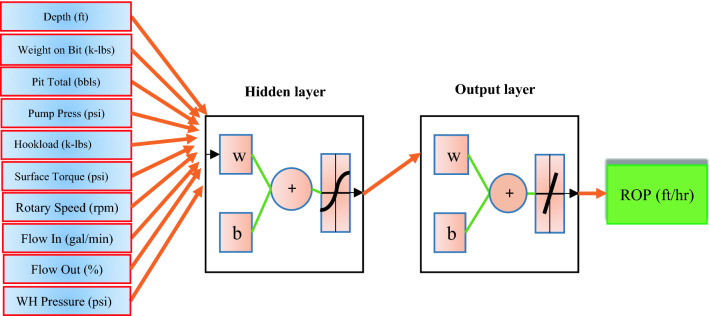
The schematic diagram of MLP.

In the present study, Purelin, Tansig, and Logsig are three-transfer function used for MLP model. As mentioned above, the first layer has a linear function and the others have nonlinear. For example, output of an MLP model with two hidden layers is calculated as follows:

26 $$output = purelin\left( {w_{3} \times \left( {tan\;sig\left( {w_{2} \times \left( {\log sig\left( {w_{1} z} \right) + b_{1} } \right)} \right) + b_{2} } \right) + b_{3} } \right)$$

where $b_{1}$, $b_{2}$, and $b_{3}$ refer to the first and second hidden layer bias vector and output layer bias, respectively. Matrixes of the first and second hidden layer and output layer are also denoted by $w_{1}$, $w_{2}$, and $w_{3}$^54,55,57,58^. Schematic of a single hidden layer MLP model illustrated in Fig. 5.

### Least square support vector machine (LSSVM)

LSSVM was firstly suggested by Suykens and Vandewalle^59^. In LSSVM, a set of linear equations is solved; therefore, we have simplification in the learning process. Eq. (27) shows the cost function of Support Vector Machine (SVM):

27 $$Cost\; function = \frac{1}{2} We^{T} We + \frac{1}{2}Tu\mathop \sum \limits_{j = 1}^{Num} Ve_{j}^{2}$$

Here superscript T represents the transport matrix of a variable and *W*e shows regression weight. A variable error of the LSSVM algorithm is shown by $Ve_{j}^{2}$ and Tu shows the tuning parameter.

Subjected to the following restriction:

28 $$Z_{j} = We^{T} \varphi \left( {y_{j} } \right) + c + Ve_{j}$$

By equating the Lagrange function of the LSSVM to zero and then using the following formula, model's parameter could be achieved:

29 $$\left\{ {\begin{array}{*{20}l} {\frac{dL}{{dWe}} = 0 \to We = \mathop \sum \limits_{j = 1}^{Num} \alpha_{j} \varphi \left( {y_{j} } \right)} \hfill \\ {\frac{dL}{{dc}} = 0 \to \mathop \sum \limits_{j = 1}^{Num} \alpha_{j} = 0} \hfill \\ {\frac{dL}{{dVe_{j} }} = 0 \to \alpha_{i} = Tu\varphi \left( {y_{i} } \right); j = 1,2, \ldots ,Num} \hfill \\ {\frac{dL}{{\alpha_{j} }} = 0 \to We^{T} \varphi \left( {y_{j} } \right) + c + Ve_{j} - Z_{j} = 0;j = 1,2, \ldots ,Num} \hfill \\ \end{array} } \right.$$

By using Eq. (31), the parameters of LSSVM can be achieved. Unknown parameters in Eq. (31), are We, c, $Ve_{j}$, and $\alpha_{j} .$

$Ve_{j}$ and $\sigma^{2}$ control the reliability of LSSVM. In this study, the amount of Tu and $\sigma^{2}$ are 24.7959, and 2.2514, respectively.

## Optimization algorithms

### Levenberg–Marquardt algorithm

In order to train data in MLP model, training algorithms are used to optimize weights and bias values. Levenberg–Marquardt is one of these algorithms which is used to solve nonlinear problems. In this method, even if there is an inappropriate initial guess for weights and bias, the algorithm will be able to get the final answer. Due to having sum square form for performance function, the gradient and Hessian matrixes are determined as follows:

30 $$g = { }J^{T} e$$

31 $$H = { }J^{T} J$$

Here, the Jacobian matrix and network errors vector are denoted by $J$ and $e$.

By updating the equations, the weight values in each step are obtained as:

32 $$w_{k + 1} = { }w_{k} - \left( {J^{T} J - \eta I} \right)^{ - 1} J^{T} e$$

It should be noted that $\eta { }$ is a constant, and due to the condition of performance function in each step, it increases or decreases^60^.

### Bayesian regularization algorithm (BRA)

Like Levenberg–Marquardt, Bayesian regularization algorithm is also used to optimize weights and bias and minimize squares of errors. Weights are determined as follows:

33 $$F\left( w \right) = \alpha E_{w} + \beta E_{D}$$

in which, $\alpha$, $\beta$, $E_{D}$, $E_{w} { }$, and *F*(*ω*) are objective function parameters, sum of network errors, sum of squared network weights, and objective function, respectively. Bayes' theorem was used to determine $\alpha$ and $\beta$ Moreover Gaussian distribution was employed to develop both network weight and training sets. These parameters are updated and repeated procedure until convergence achieved^61^.

### Boosting method

Schapire^62^ introduced boosting method which is a type of ensemble methods. In this method, some weak predictors/learners are combined to create a stronger learner. In order to correct previous learners, each weak learner is trained. One of the most popular types of Boosting is Gradient Boosting which is used in this paper.

#### Gradient boosting (GB)

Gradient boosting is known as one type of Boosting methods. In this type, new learners are applied to residual errors which are made by the previous learners^63^. The GB could be considered as a form of functional gradient decent (FGD), in which a specific loss is lessened by adding a learner at each step of gradient descent^64^. The algorithm of GB is as follows:

1. Initialize $$g_{0} \left( y \right) = argmin_{\gamma } \mathop \sum \limits_{q = 1}^{Nu} O\left( {x_{q} ,\gamma } \right)$$
2. Iteration for c = 1: C (C is number if tree learners
  - Compute the negative gradient $a_{q} = \left[ {\frac{{\partial O(x_{q} ,g\left( {y_{q} } \right)}}{{\partial g\left( {y_{q} } \right)}}} \right]_{{g = g_{c - 1} }} ,{ }q = 1,2, \ldots ,NU$
  - Set a regression free $F_{c} \left( y \right)$ to the target $\left\{ {a_{q} ,{ }q = 1,2, \ldots ,NU} \right\}$
  - Compute the gradient descent step size by following equation:
    $$t = argmin_{\gamma } \mathop \sum \limits_{q = 1}^{Nu} O\left( {x_{q} ,g_{c - 1} \left( {y_{q} } \right) + \gamma F_{c} \left( {y_{q} } \right)} \right)$$
  - Update the model as $g_{c} \left( y \right) = g_{c - 1} \left( y \right) + tF_{c} \left( y \right)$
3. For data test (y,?) output is $g_{C} \left( y \right)$

The parameters of GB used in this study are presented in Table 1.

**Table 1:** The parameters used in Gradient Boosting trees.

Number of features to consider when looking for the best split	10
Fraction of samples to be used for fitting the individual base learners	0.8
Minimum number of samples required to split an internal node	2
Maximum depth of the individual regression estimators	6
Minimum number of samples required to be at a leaf node	3
Number of boosting stages to perform	140

## Results and discussion

**Figure 6:**
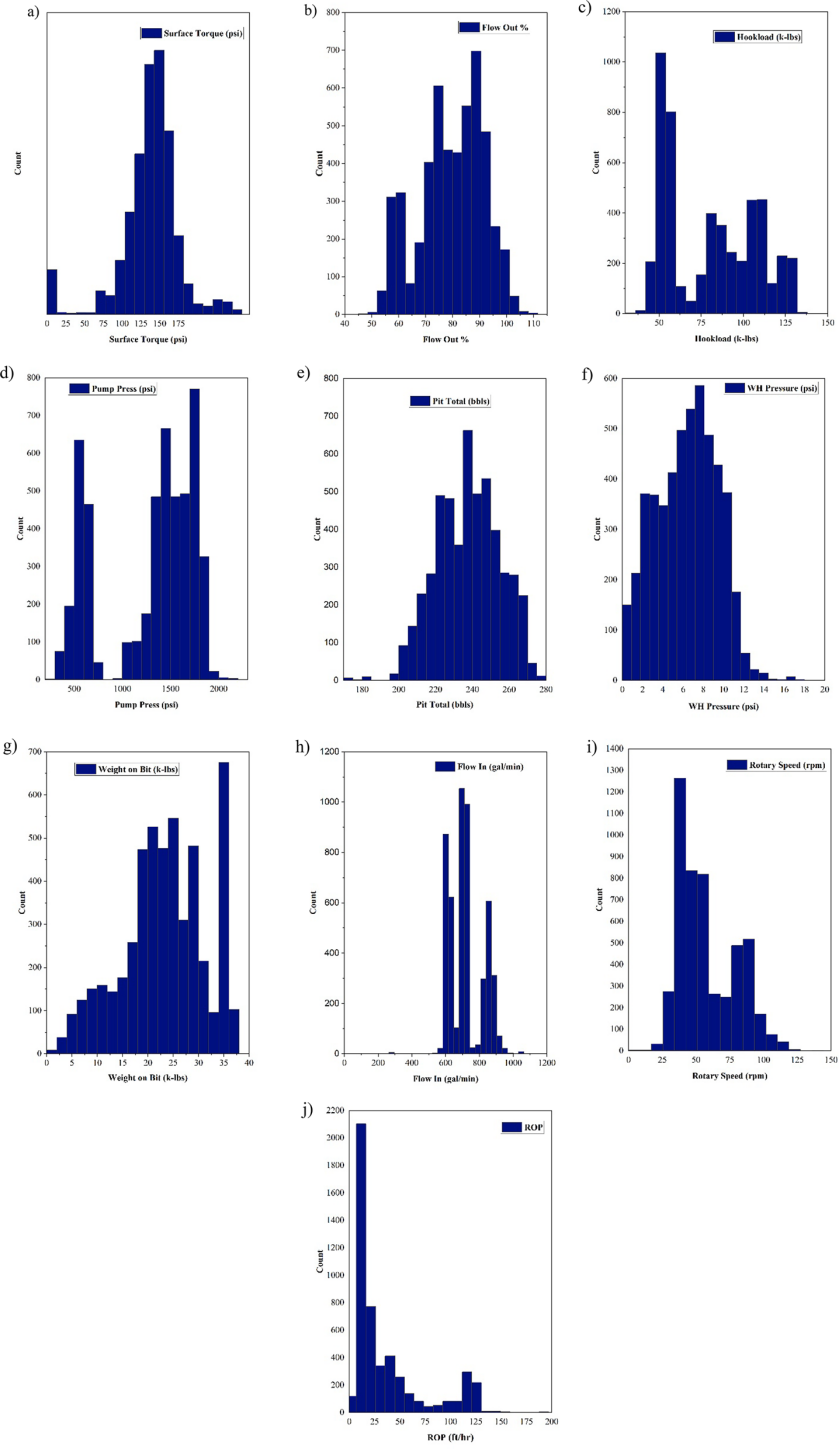
Histogram of inputs and output data.

**Figure 7:**
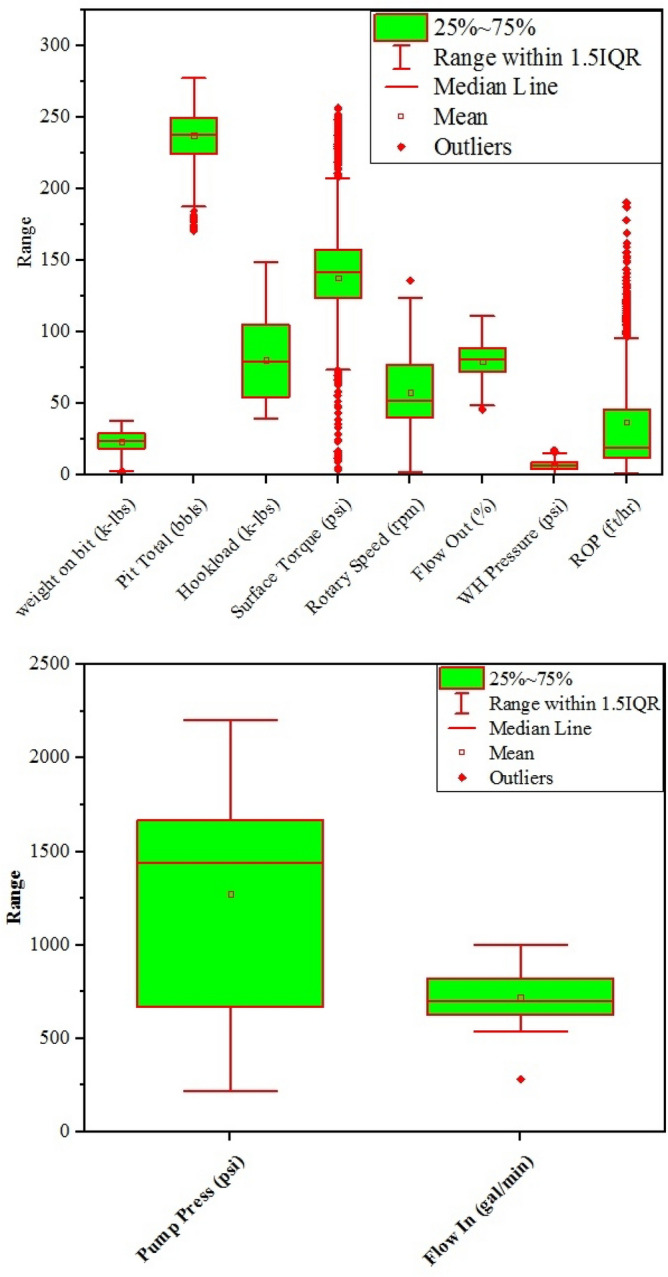
Box plot of inputs and output data.

**Figure 8:**
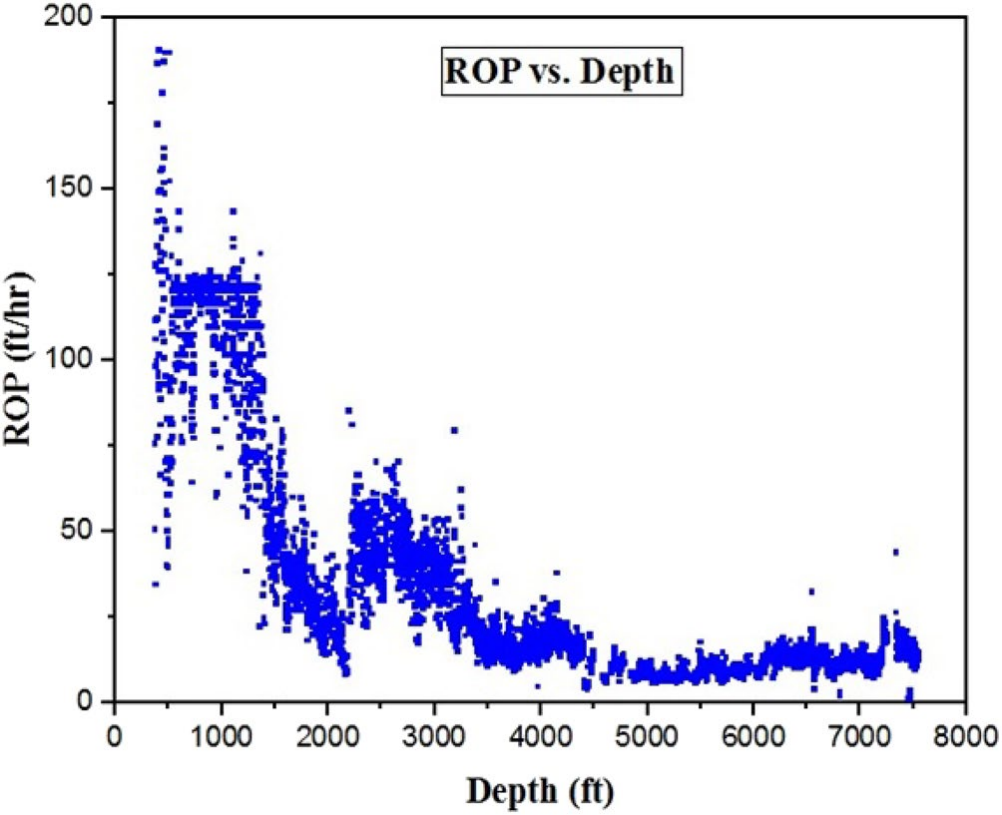
The relation between ROP and depth.

In this research, 5040 data points from South Azadgan field in Iran have been used. Table 2 shows the preprocessing of this dataset. In all the developed models, depth (D), weight on bit (WOB), pit total (PT), pump pressure (PP), hook load (H), surface torque (ST), rotary speed (RS), flow in (Fi), flow out (Fo), and wellhead pressure (Wp) were considered as inputs and ROP is regarded as output. Histogram of inputs and output are presented in Fig. 6. As shown in Fig. 6 most of data of surface torque are between 75 and 175 psi. Figure 6 showed that data of flow out and flow in are altered between 50–100% and 600–800 gal/min, respectively (Fig. 6). Hook load data varied from 75–125 k-lbs and most of them are 50 k-lbs (Fig. 6). Data of pump pressure and wellhead pressure are varied from 1000 to 2000 psi and from 0 to 10 psi, respectively (Fig. 6). Pit total data lie between 200 and 280 bbls (Fig. 6). Most of Weight on bit data are around 35 k-lbs (Fig. 6). Most of the rotary speed data in our study were from 25 to100 rpm. Maximum ROP in our data is around 25 ft/hr (Fig. 6). Figure 7 shows box plot of inputs and output data. As shown in Fig. 7, range of WOB data are lower than 50, while pit total data are higher than 200. The data of hook load varied between 50 and 100 (Fig. 7). The range of surface torque and rotary speed are less than 150 and 100, respectively. In addition, the range of flow out and wellhead pressure are less than 100 and 25, respectively (Fig. 7). As shown in Fig. 7, 25% to 75% of ROP's data are less than 50. Figure 7 shows that pump pressure is varied from 750 to more than 1500. As stated in Fig. 7, all of the flow in data are less than 1000. Figure 8 shows the relation of ROP vs. depth for our data. As shown in Fig. 8, by increasing the depth, ROP will decrease.

**Table 2:** Statistical parameters of the inputs and output data.

	Depth (ft)	WOB (k-lbs)	PT (bbls)	PP (psi)	H (k-lbs)	ST (psi)	RS (rpm)	Fi (gal/min)	Fo (%)	Wp (psi)	ROP (ft/hr)
Mean	3797.92	23.29	237.52	1274.14	80.18	137.92	57.77	720.48	79.59	6.38	37.36
Median	3617.93	23.65	238.07	1441.28	79.16	0.54	51.94	702.07	81.03	6.51	19
Mode	1130.84	24.52	247.17	505.28	51.71	141.57	50.38	727.37	73.83	8.26	120.4
SD	2118.30	8.23	17.07	482.25	26.59	38.93	21.39	104.45	11.95	2.97	37.74
Kurtosis	−1.24	−0.45	−0.29	−1.14	−1.34	3.55	−0.2	74.76	−0.65	−0.53	2.49
Skewness	0.12	−0.34	−0.18	−0.61	0.27	−0.99	0.7	3.35	−0.36	−0.016	1.63
Minimum	346.63	0.40	170.91	220.48	39.42	3.56	1.69	280.87	45.67	0.03	1.11
Maximum	7533.96	37.89	277.64	2200.43	148.93	257.09	207.23	3317.51	111.21	17.41	190.78

In first step, in order to estimate ROP based on input parameters, the following correlation was developed based and its coefficients were optimized by GRG:

34 $$\begin{aligned} {\text{ROP}} &= a_{1} + \left( {a_{2} *D} \right) + \left( {a_{3} *Wp} \right) + \left( {a_{4} *Fo} \right) + \left( {a_{5} *Fi} \right) + \left( {a_{6} *RS} \right) \\ &\quad + \left( {a_{7} *ST} \right) + \left( {a_{8} *H} \right) + \left( {a_{9} *PP} \right) + \left( {a_{10} *PT} \right) + \left( {a_{11} *WOB} \right) + \left( {\frac{{a_{12} }}{D}} \right) \\ &\quad + \left( {\frac{{a_{13} }}{WOB}} \right) + \left( {\frac{{a_{14} }}{PT}} \right) + \left( {\frac{{a_{15} }}{PP}} \right) + \left( {\frac{{a_{16} }}{H}} \right) + \left( {\frac{{a_{17} }}{ST}} \right) + \left( {\frac{{a_{18} }}{RS}} \right) + \left( {\frac{{a_{19} }}{Fi}} \right) + \left( {\frac{{a_{20} }}{Fo}} \right) \\ &\quad + \left( {\frac{{a_{21} }}{Wp}} \right) + \left( {a_{22} *\ln \left( {Wp} \right)} \right) + \left( {a_{23} *\ln \left( {Fo} \right)} \right) + \left( {a_{24} *\ln \left( {Fi} \right)} \right) \\ &\quad + \left( {a_{25} *\ln \left( {RS} \right)} \right) + \left( {a_{26} *\ln \left( {ST} \right)} \right) + \left( {a_{27} *\ln \left( H \right)} \right) + \left( {a_{28} *\ln \left( {PP} \right)} \right) \\ &\quad+ \left( {a_{29} *\ln \left( {PT} \right)} \right) + \left( {a_{30} *\ln \left( {WOB} \right)} \right) + \left( {a_{31} *\ln \left( D \right)} \right) \\ \end{aligned}$$

where a_1_–a_31_ are constants which are presented in Table 3. As shown in Eq. (34), all involved parameters are available and recorded during drilling operation. Therefore, this correlation can be used to estimate ROP roughly. Although the developed correlation can give us a good sense of ROP, if we want to have a good estimation of ROP, it is recommended to use artificial intelligence (AI) which are more flexible and could solve complicated problems. In this study, AI methods namely, LSSVM, MLP, RBF, and DT were used. In order to develop AI models, first, the databank was randomly separated into two subgroups known as the training set, in which the model learns and tries to find best and optimum predictive model, and the test set, which is used to investigate the prediction capability of the developed model. Classification of data points for intelligent models and the developed correlation are as follow:

1. 80 percent of the data were used for training
2. 20 percent of the data were used for testing

**Table 3:** The constants of developed correlation.

Constants	Value
a_1_	0.0746091
a_2_	0.0192657
a_3_	0.3659792
a_4_	0.6389874
a_5_	−0.043096
a_6_	−0.157758
a_7_	0.0211645
a_8_	−0.044939
a_9_	−0.027585
a_10_	−0.066651
a_11_	0.1685122
a_12_	8740.1755
a_13_	33.571705
a_14_	1.0766685
a_15_	0.3168871
a_16_	352.91161
a_17_	−98.26846
a_18_	60.965293
a_19_	−0.009591
a_20_	8398.8922
a_21_	−0.021988
a_22_	−1.244715
a_23_	59.887972
a_24_	0.0048745
a_25_	13.832765
a_26_	−2.767219
a_27_	1.7682843
a_28_	52.402178
a_29_	5.6629656
a_30_	1.101E−05
a_31_	−103.9342

LMA, BR, and SCG are the three algorithms developed for MLP model and GB is the optimization technique used for DT model.

### Statistical evaluation

In order to evaluate and compare the developed models in this study, statistical analysis of errors is performed. For this purpose, the values of standard deviation (SD), average absolute percent relative error (AAPRE), coefficient of determination (R^2^), root mean square error (RMSE), and the average percent relative error (APRE) are computed and the results are summarized in Table 4. Equations (35)–(39) presented the formulation employed to calculate the aforementioned parameters^58,65^.

35 $$SD = \sqrt {\frac{1}{Num}\mathop \sum \limits_{l = 1}^{Num} \left( {\frac{{ROP_{exp,l} - ROP_{pred,l} }}{{ROP_{exp,l} }}} \right)^{2} }$$

36 $$R^{2} = 1 - \frac{{\mathop \sum \nolimits_{l = 1}^{Num} \left( {ROP_{exp,l} - ROP_{pred,l} } \right)^{2} }}{{\mathop \sum \nolimits_{l = 1}^{Num} \left( {ROP_{pred,l} - \overline{ROP} } \right)^{2} }}$$

37 $$AAPRE = \frac{100}{{Num}}\mathop \sum \limits_{l = 1}^{Num} \frac{{\left| {ROP_{exp,l} - ROP_{pred,l} } \right|}}{{ROP_{exp,l} }}$$

38 $$RMSE = \sqrt {\frac{1}{Num}\mathop \sum \limits_{l = 1}^{Num} \left( {ROP_{exp,l} - ROP_{pred,l} } \right)^{2} }$$

39 $$APRE = \frac{100}{{Num}}\mathop \sum \limits_{l = 1}^{Num} \frac{{ROP_{exp,l} - ROP_{pred,l} }}{{ROP_{exp,l} }}$$

**Table 4:** Statistical error analysis of the developed models for the rate of penetration.

Developed models	R^2^	APRE %	RMSE	AAPRE %	SD
**The developed correlation**					
Train	0.814	3.963	15.904	22.791	0.299
Test	0.837	3.701	15.170	22.475	0.291
Total	0.807	4.000	16.559	23.556	0.365
**MLP-LMA**					
Train	0.971	−1.838	6.21	13.762	0.191
Test	0.941	−2.539	9.056	15.247	0.235
Total	0.965	−1.978	6.873	14.059	0.201
**MLP-BRA**					
Train	0.973	−0.665	5.943	13.661	0.186
Test	0.953	−1.316	8.135	14.772	0.227
Total	0.969	−0.795	6.441	13.883	0.195
**MLP-SCGA**					
Train	0.944	−5.205	8.664	18.551	0.355
Test	0.945	−6.08	8.919	18.263	0.27
Total	0.944	−5.38	8.716	18.493	0.34
**RBF**					
Train	0.94	−3.733	9.09	21.261	0.347
Test	0.928	−3.379	9.731	22.003	0.321
Total	0.937	−3.663	9.221	21.409	0.342
**LSSVM**					
Train	0.975	−1.971	5.821	10.023	0.14
Test	0.956	−2.535	7.656	12.394	0.168
Total	0.971	−2.084	6.231	10.497	0.146
**DT-GB**					
Train	0.978	−1.083	5.413	8.343	0.139
Test	0.97	−1.727	6.346	11.707	0.168
Total	0.977	−1.211	5.611	9.013	0.145

Two suitable statistical errors to compare the developed models are AAPRE and R^2^. As presented in Table 4, R^2^ of the developed correlation is 0.807. Then, among different MLP models, the best performance was for BRA, followed by LMA and SCGA. R^2^ of MLP-SCGA, MLP-LMA, and MLP-BRA were 0.944, 0.965, and 0.969, respectively. AAPRE of these models is in good agreement with the R^2^ results, 13.88% for MLP-BRA, 14.05% for MLP-LMA, and 18.49% for MLP-SCGA. As shown in Table 4, RBF had the worst performance among the developed models. AAPRE and R^2^ of this model are 21.409% and 0.937, respectively. R^2^ and AAPRE for LSSVM are 0.971 and 10.497%, respectively. As stated in Table 4, DT-GB had the best performance among the developed models. AAPRE for this model is 9.013% and its R^2^ is 0.977. Therefore, DT-GB has the best performance among the developed models, followed by LSSVM, MLP-BR, MLP-LM, MLP-SCG, and RBF.

### Graphical analysis of models

**Figure 9:**
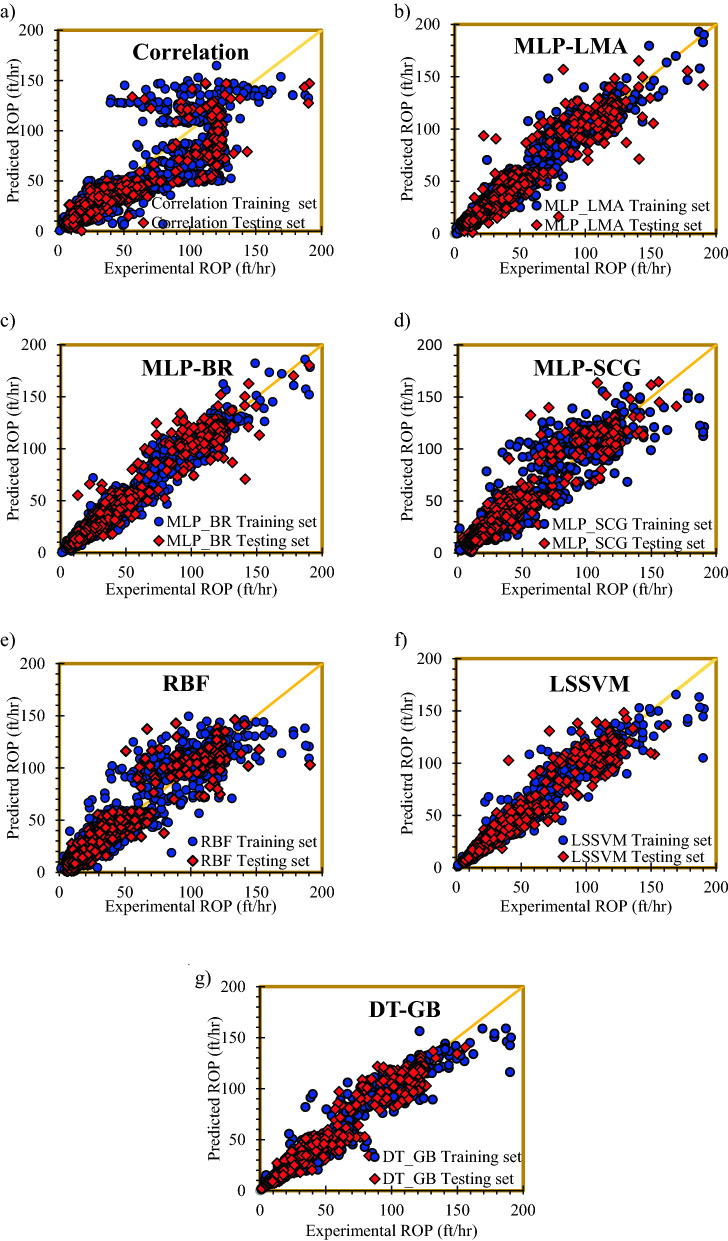
Cross plots of the implemented intelligent models.

Figure 9 shows the crossplots for the developed models. In these plots, the values of modeled ROP are plotted versus experimental data. The more data around the line Y = X is, the more accurate the model will be. In other words, line Y = X is a visual criterion for quick examination of model accuracy. Parameter R^2^ specifies how much data sets conform to the line of Y = X. In other words, as far as R^2^ is closer to 1, the degree of conformance of the model with the experimental data is more remarkable. Subplot (a) of Fig. 9 presents crossplots of the developed models. As shown in subplot (a), until ROP of 50, the developed correlation obtains an acceptable prediction. However, at high ROP values, scattering of data around 45^o^ line is obvious. As shown in subplot (b) of Fig. 9, except at high ROP values, concentration of the data around the unit slope line is well for MLP-LMA. Concentration of training set around the unit slope line is better than testing set in MLP-LMA model. The same results were achieved for MLP-BRA; however, a better concentration of the data is noticed in MLP-BRA than MLP-LMA (subplot (c) of Fig. 9). However, scattering of data is obvious for MLP-SCGA (subplot (d) of Fig. 9). Scattering of the testing set is obvious and much more than the training data. In subplot (e) of Fig. 9, it can be seen that the estimations of RBF model are scattered around the Y = X line. Scattering of the testing data both at high and low ROP values is obvious. Although scattering of the test data is obvious, concentration of the training data around the Y = X line is acceptable for LSSVM (subplot (f) of Fig. 9. Subplot (g) of Fig. 9 shows that the best performance among AI models belongs to DT-GB. As shown in subplot (g) of Fig. 9, concentration of the data around 45° straight line is good.

**Figure 10:**
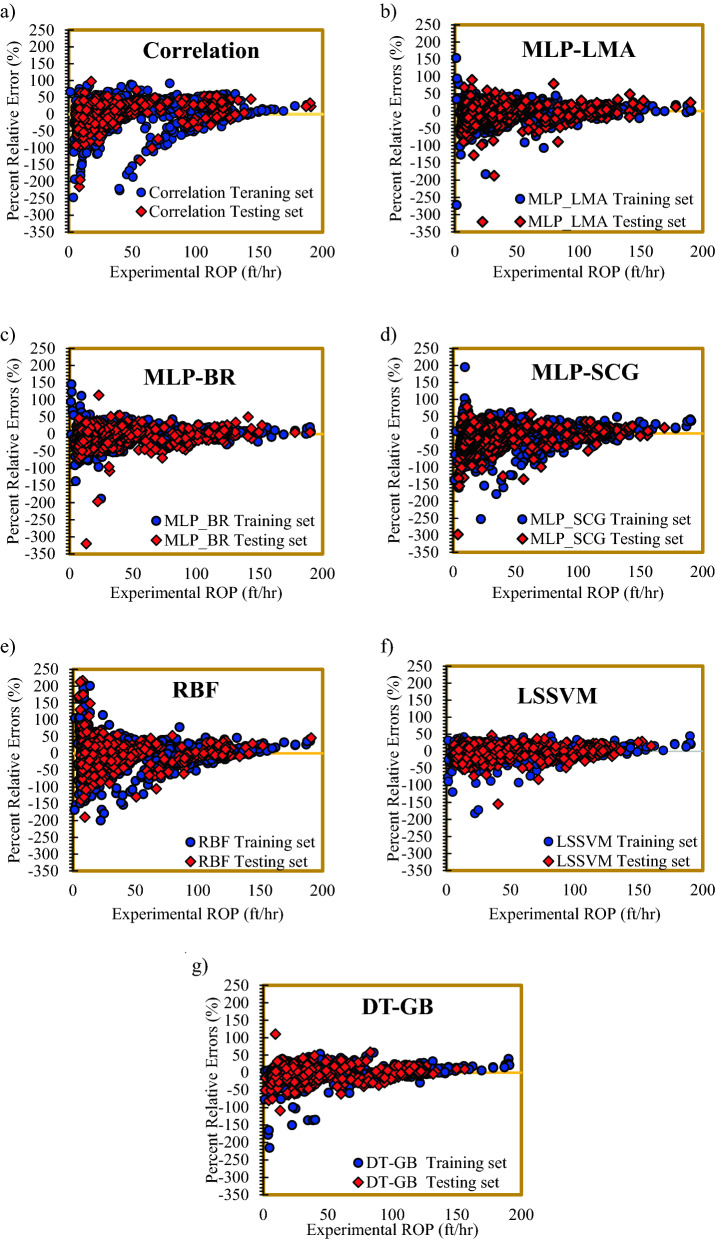
Error distribution plots of the proposed models.

Error distribution of the proposed correlation and developed models is presented in Fig. 10. In each subplot, the percent relative error is plotted against rate of penetration. Subplot (a) of Fig. 10 shows that the developed correlation has reasonable prediction at low ROP values and concentration of the data points around the zero-error line is good. As shown in subplot (b) of Fig. 10, concentration of the data sets around the zero-error line is suitable. In addition, subplot (c) of Fig. 10 shows a much better concentration of the data for MLP-BRA around the zero-error line than MLP-LMA. However, concentration of the data points, which are estimated by model MLP-SCGA, around zero-error line is not as good as that of the two other MLP models (subplot (d) of Fig. 10). Statistical analysis showed that the performance of RBF is not well. Both cross plot and error distribution of RBF confirmed this finding (subplot (e) of Fig. 10). As illustrated in subplot (f) of Fig. 10, concertation of the training data around the zero-error line is satisfactory for LSSVM model, although concentration of the testing data was not well at some points. As displayed in subplot (e) of Fig. 10, the predictions of DT-GB display very appropriate concentration around the zero-error line at both high and low ROP values. The subplot (e) of Fig. 10 supports the superiority of DT-GB.

**Figure 11:**
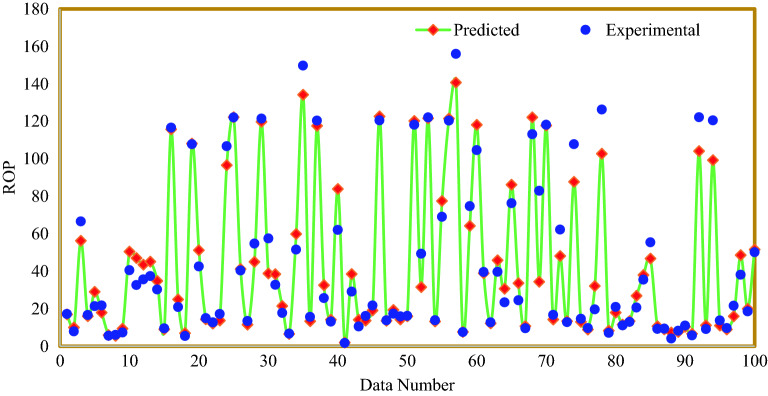
Comparison of experimental data and output of DT-GB model for the first 100 testing data points.

Figure 11 shows comparison between experimental ROP and ROP predicted values by DT-GB model for the first 100 testing data points. As shown in Fig. 11, the best developed model in this study, DT-GB, has good predictions. Except in some data points, the predictions of DT-GB match well with the experimental ROP.

**Figure 12:**
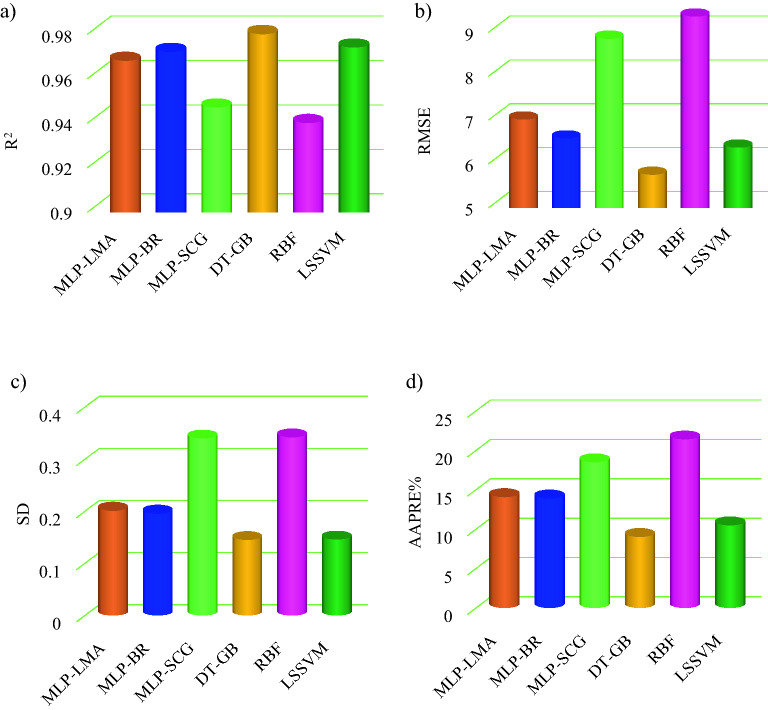
Comparison of statistical errors of the intelligent models.

Figure 12 shows the comparison of statistical errors for developed models using bar chart. Each subplot of Fig. 12 confirms that the best and worst performance belong to DT-GB and RBF, respectively.

**Figure 13:**
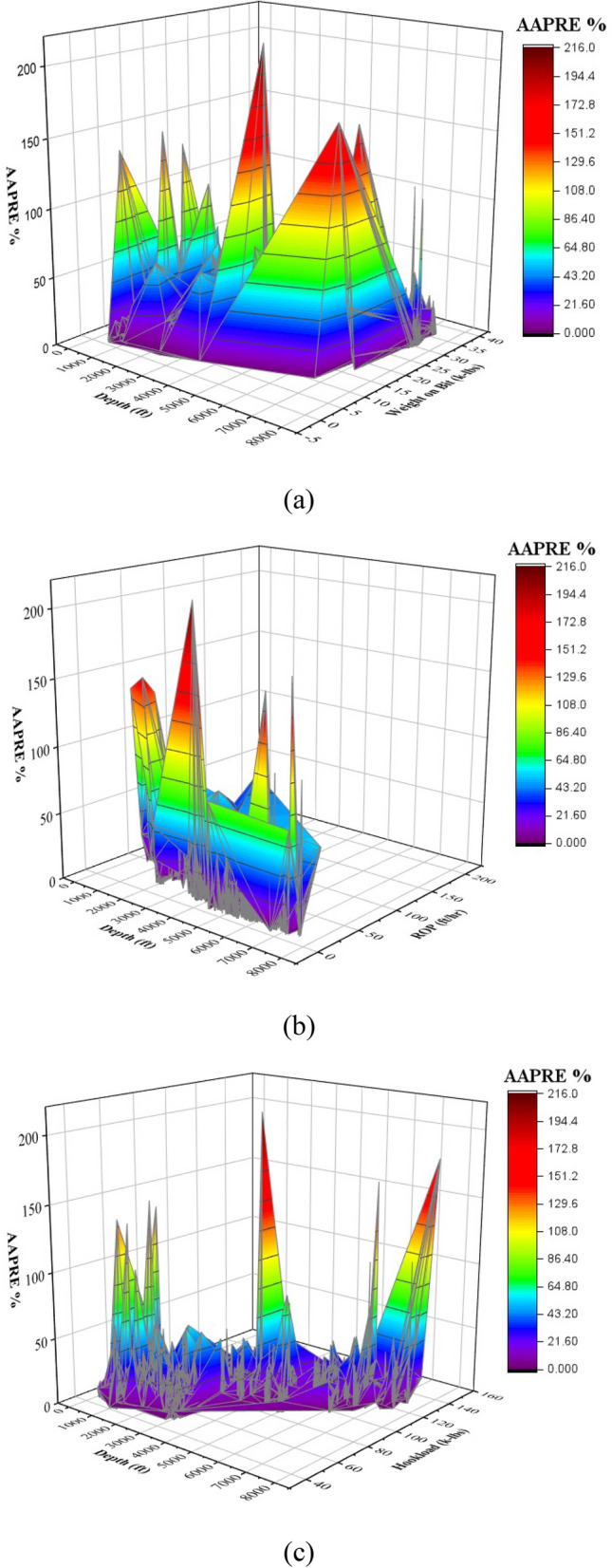
Absolute relative error contour versus different parameters (**a**) WOB and depth (**b**) ROP and depth (**c**) hook load and depth.

3D plot of absolute relative error of DT-GB model versus different parameters including, hook load, depth, ROP, and WOB, are shown in Fig. 13. As shown in subplot (a) of Fig. 13, maximum absolute relative error is seen when WOB is around 18 k-lbs and depth is 4000 ft. In subplot (b) of Fig. 13, once ROP is 14 ft/hr, and depth is 4000 ft, maximum absolute relative error is reported. Also, at Hook load 90 k-lbs and depth of 4000 ft, the model has high error.

**Figure 14:**
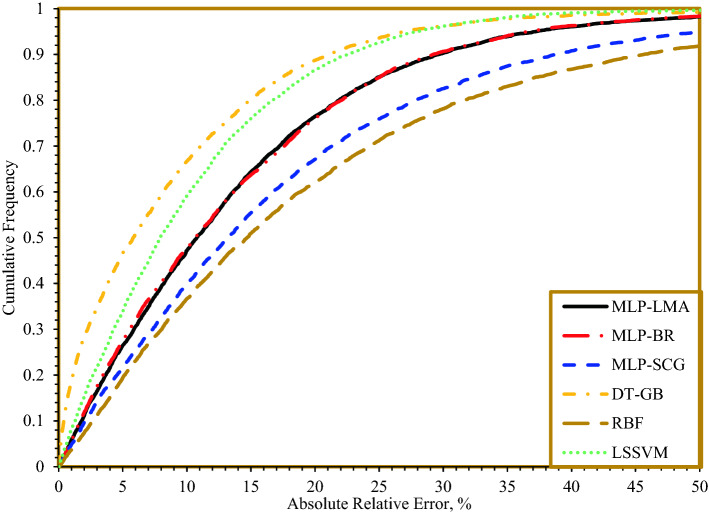
Cumulative frequency vs. absolute relative error of different models proposed in this study.

Figure 14 shows cumulative frequency vs. absolute relative error. Above 50% of the predicted ROP values by DT-GB models have an absolute relative error of less than 10%. 50% of the predicted ROP by LSSVM have an error less than 10%. About 50% of the predicted values by MLP-LMA and MLP-BR models have an absolute relative error of less than 10%. For MLP-SCG and RBF, about 40% and around 30% of the predicted ROP values, respectively, have an absolute relative error of less than 10%.

### Sensitivity analysis

A sensitivity analysis was investigated to study the quantitative effects of all input parameters on the ROP of the developed model. Relevancy factor with directionality (*r*) was chosen for this purpose. The value of r and its sign show the level of effect of input on the output of model and the impact direction, respectively^66^. The following formula shows the definition of r:

40 $$r\left( {In_{n} ,OU} \right) = \frac{{\mathop \sum \nolimits_{j = 1}^{m} (In_{n,j} - \overline{{In_{n} )}} \left( {OU_{j} - \overline{Ou} } \right)}}{{\sqrt {\mathop \sum \nolimits_{j = 1}^{m} (In_{n,j} - \overline{{In_{n} )}}^{2} \mathop \sum \nolimits_{j = 1}^{m} (Ou_{j} - \overline{Ou)}^{2} } }}$$

**Figure 15:**
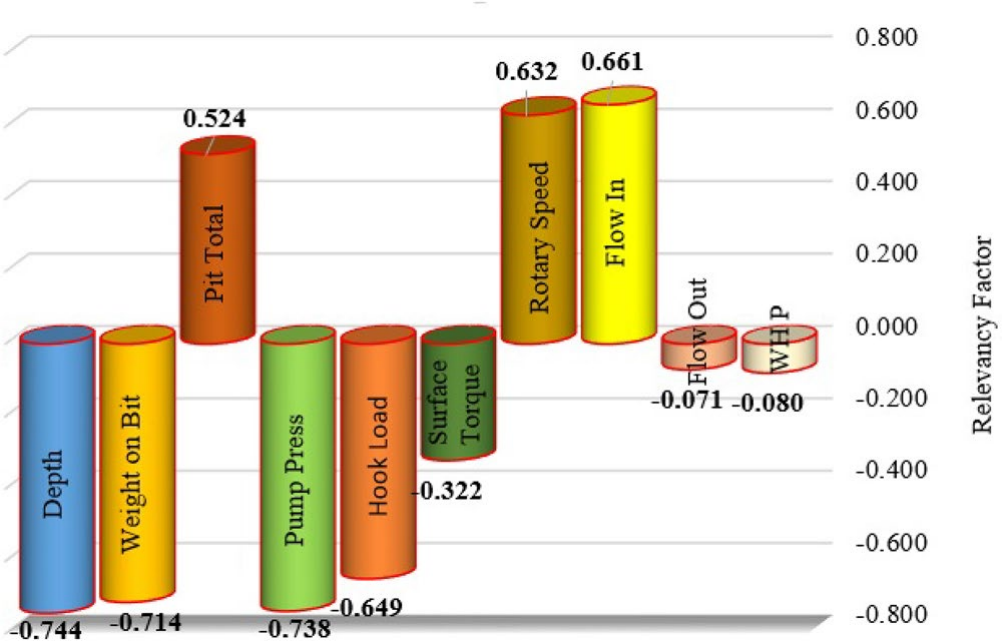
The relative effect of input variables on the ROP based on DT-GB model.

In the above equation, $In_{k}$ and $OU$ show the nth input of the model and the predicted ROP, respectively. The relative effect of input variables on the ROP estimated by the proposed DT-GB model is shown in Fig. 15. As shown in Fig. 15, pit total, rotary speed, and flow in, have a positive effect on the ROP, while depth, weight on bit, pump pressure, hook load, surface torque, and wellhead pressure have negative impacts on the ROP. The highest absolute value of r belongs to depth; therefore, depth has the most important effect among the inputs on the predicted ROP value.

### Applicability area of the developed model and outlier analysis

Outliers are the data that may vary from the bulk of the data. Frequently, these types of data are expected to appear in large sets of experimental data. The presence of such data can affect the accuracy and reliability of models. Hence, finding these data is necessary in the development of models^67–71^. In this study, leverage approach has been employed for determining outliers^67,69–71^. In this method, deviation of predicted valued from corresponding experimental data, was calculated. More details about this method can be found in literature^67–70^.

**Figure 16:**
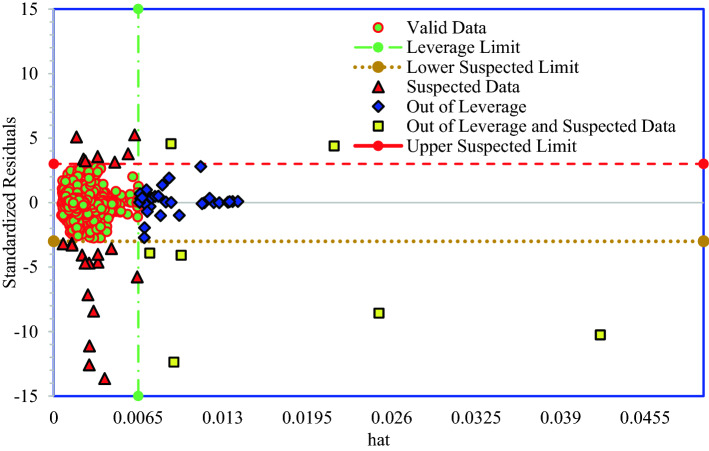
William's plot for discovering the probable outliers and the feasibility domain of the developed model, DT-GB, in this study.

Figure 16 shows the William's plot for the predicted ROP obtained by the DT-GB model. Data of out of leverage and suspected data, presented in Fig. 16, can be found in Table 5. As shown in Fig. 16, majority of the data points are positioned in the applicability domain (− 3 ≤ R ≤ 3 and 0 ≤ hat ≤ 0.0057). Therefore, the developed model, DT-GB, has statistical validity and high reliability. A small amount of data is out of the applicability domain, which is negligible. In this plot, we have two important definitions, Good High Leverage and Bad High Leverage. Good High Leverage data are known as data that their R is located between 3 and -3 and their hat* ≤ hat. These data points are different from the bulk of data and they are out of feasibility domain of the developed model, however, they may be predicted well by developed model. If R of data are less than -3 or are more than 3, these data are known as Bad High Leverage. These data are experimentally doubtful data or outliers^67–70^.

**Table 5:** Inputs and output which are out of the applicability domain of the DT-GB model based on the leverage approach.

No	Depth (ft)	WOB (k-lbs)	PT (bbls)	PP (psi)	H (k-lbs)	ST (psi)	RS (rpm)	Fi (gal/min)	Fo (%)	Wp (psi)	ROP(ft/hr)
**Suspected data**											
1	1196.45	15.52	257.84	553.69	48.78	139.36	84.15	682.15	61.36	2.16	89.31
2	5944.05	34.7	237.21	1335.59	104.04	194.95	36.81	601.07	70.67	3.08	10.35
3	6059.11	35.89	236.42	1430.29	99.02	170.01	39.02	595.03	74.39	9.94	12.08
4	2749.46	18.92	266.26	1504.55	59.48	154.65	52.33	693.87	97.16	2.54	40.03
5	741.772	4.86	258.13	536.54	54.64	113.62	71.25	912.48	63.53	7.88	122.11
6	5916.79	35.29	245.31	1400.74	100.8	151.44	39.72	586.02	71.05	7.65	8.94
7	6062.13	35.68	236.31	1409.07	99.23	177.83	32.85	585.76	73.87	9.94	10.32
8	5440.73	35.51	250.17	1729.19	105.83	70.24	35.49	657.32	82.77	5.56	7
9	6133.83	34.96	232.53	1461.35	104.17	214.35	40.92	594.17	73.97	1.17	12.28
10	2421.76	21.64	231.55	1094.08	53.17	163.45	56.73	692.66	102.28	10.55	46.78
11	2925.46	23.4	249.75	1604.92	55	146.41	52.64	692.66	93.54	5.97	37.18
12	670.957	5.79	259.83	532.73	55.51	64.4	97.27	902.01	61.33	7.88	120.41
13	4644.25	10.55	228.27	2015.57	105.74	152.13	52.7	714.44	88.61	0.61	10.42
14	7181.33	30.32	220.82	1574.99	120.37	133.34	37.79	626.5	74.06	5.74	13.66
15	5332.56	31.12	248.61	1688.28	104.12	104.93	26.22	673.49	79.85	5.56	11.92
16	1663.03	23.39	231.47	624.99	49.29	149.81	79.24	847.14	80.95	8.64	38.92
17	5505.55	24.14	234.91	1220.82	110.51	193.15	75.84	640.29	71.8	4.6	9.59
18	1710.88	23.57	240.49	606.31	51.13	196.2	86.32	836.69	78.62	5.59	44.11
19	380.874	6.34	275.2	327.61	78.66	4.74	79.27	864.4	59.02	3.69	100
20	1102.67	10.41	259.15	530.44	53.89	152.74	111.16	659.34	55.18	3.31	89.35
21	941.294	14.8	247.16	514.04	45.6	229.98	76.04	824.25	61.63	7.88	60.11
22	500.889	10.04	250.87	414.54	49.36	4.82	101.3	803.94	58.14	5.59	215.04
**Out of leverage**											
23	853.358	11.37	253.11	505.28	49.03	145.08	79.3	858.05	60.83	8.64	120.4
24	1860.91	20.49	240.43	641.38	54.21	90.71	99.07	871.04	90.59	0.64	32.83
25	3263.37	24.91	243.82	1586.06	78.29	158.76	37.72	688.55	89.74	7.5	41.43
26	3049.09	28.11	240.88	1566.41	54.29	146.41	71.13	694.09	97.8	10.93	21.86
27	2082.37	26	236.94	720.3	50.26	127.96	103.41	848.3	89.84	6.36	17.21
28	1160.66	12.47	265.87	565.13	51.83	143.51	77.72	858.25	55.59	3.31	115.24
29	837.909	5.21	243.45	549.5	55.09	75.5	95.44	895.53	62.53	8.26	122.11
30	6926.93	23.5	213.23	1496.2	127.24	142.94	34.88	607.58	77.55	0.41	12.11
31	3294.01	21.61	223.57	1668.94	78.79	110.61	29.54	695.51	79.74	5.21	30.33
32	7429.92	35.7	230.53	1712.88	120.19	145.92	34.17	613.58	75.38	11.08	18.92
33	2502.31	16.94	246.82	1421.91	58.11	149.46	54.56	697.76	98.46	10.93	40.26
34	660.69	3.42	259.87	542.26	57.88	64.75	90.44	903.14	61.92	7.88	122.11
35	5316.36	29.46	245.99	1401.14	104.98	103.22	29.82	620.25	73.83	7.09	8.13
36	681.256	6.23	258.86	560.56	55.07	70.16	78.42	927.25	61.48	7.88	124.41
37	6561.84	30.2	215.77	1798.49	113.84	145.5	48.36	726.73	85.69	6.51	8.3
38	1760.77	23.86	245.23	625.75	50.84	145.27	87.54	828.01	87.47	3.69	41.43
39	3559.03	28.3	223.52	1784.94	72.1	136.31	37.63	718.1	85.59	9.79	18.6
40	3597.18	28.63	226.84	1751.07	71.77	150.07	60.54	726.1	89.74	9.41	19.84
41	2835.4	20	261.94	1491.74	58.4	152.63	54.16	693.27	90.42	2.54	39.64
42	4691.78	17.5	246.46	1797.37	94.29	180.19	43.48	712.22	80.09	4.42	11.8
43	2671.79	22.49	257.61	1472.35	55.91	145	52.55	688.14	99.65	4.83	35.66
44	544.086	7.73	258.59	475.92	52.04	4.7	85.92	906.97	56.47	5.59	122.13
45	3032.75	28.07	237.82	1575.3	54.33	160.21	64.05	691.22	94.84	8.64	42.45
46	3275.41	26.82	244.62	1575.61	76.38	164.45	52.91	691.41	93.4	7.12	24.72
47	7199.53	19.38	204.93	1706.06	137.39	144.62	42	614.03	66.86	5.74	13.14
48	6226.19	35.39	217.17	1320.43	97.13	154.42	44.54	609.71	77.31	10.7	16.04
49	6363.59	35.24	227.72	1284.07	109.27	123.73	47.46	602.17	73.64	9.18	13.37
50	5621.3	29.87	237.62	1347.35	104.85	153.5	87.14	653.44	71.94	7.65	10.3
51	3914.84	27.1	243.66	1826.82	80.19	127.01	45.44	726.87	88.19	6.74	19.39
52	373.002	27.31	275.21	327.23	57.69	4.74	84.25	862.77	59.99	3.31	98.48
53	3770.06	2.95	223.82	1802.58	101.34	98.76	119.45	732.04	95.85	5.59	21.54
**Suspected data and out of leverage**											
54	6428.44	34.97	228.6	1320.43	111.25	136.69	51.78	612.06	70.95	6.51	13.93
55	932.11	16.69	250.17	507.94	43.71	181.72	76.62	855.8	61.07	7.5	114.49
56	1062.42	11.85	250.96	543.02	52.45	226.32	82.66	891.65	58.14	5.59	120.51
57	3904.84	28.85	246.83	1810.91	78.44	128.38	53.06	726.65	86.54	7.12	15.35
58	2664.74	21.25	257.16	1483.91	57.15	147.82	51.05	693.26	95.34	4.83	44.25
59	7177.3	30.37	221.49	1536.35	120.33	132.5	37.33	633.18	73.69	6.13	14.66
60	537.92	9.27	258.68	468.29	50.5	4.66	72.99	904.3	60.83	5.21	120.4

## Conclusions

In this study, new methods were used to predict drilling rates. Since the parameters affecting the drilling rates are different, as well as the conditions vary from field to field, it is always difficult to develop a comprehensive, efficient, and precise model. The model that can accommodate more parameters, could better predict the drilling rate. Therefore, we tried to develop a correlation and smart models including MLP, RBF, LSSVM, and DT, with ten input parameters. The main findings of this study are as follows:

1. The developed correlation and smart models need parameters that are accessible in field and can give fast prediction of ROP.
2. All four smart models have a good prediction of drilling rates, which would increase the tendency to use smart methods to predict drilling rates.
3. The best predictions belong to DT-GB model with R^2^ of 0.977. In addition, the LSSVM model has acceptable performance. R^2^ of this model was 0.969. In addition, MLP models have good performance and finally the worst performance among the developed models belongs to RBF.
4. Sensitivity analysis showed that flow in, rotary speed, and pit total have positive effects on ROP, while other parameters have negative effects. Among input parameters, depth has the greatest effect on ROP.
5. The leverage approach indicated that the developed DT-GB model is statistically valid and only few data points are located out of the applicability domain of the model.

## Abbreviations

RBF: Radial basis function
DT: Decision tree
MLP: Multilayer perceptron
LMA: Levenberg_Marquardet algorithm
BRA: Bayesian regularization algorithm
SCGA: Scaled conjugate gradient algorithm
GB: Gradient boosting
APRE: Average percent relative error
AAPRE: Average absolute relative error
RMSE: Root mean square error
SD: Standard deviation
ML: Machine learning
BYM: Bourgoyne and Yong model
WOB: Weight on bit
ANN: Artificial neural network
D: Depth
PT: Pit total
PP: Pump pressure
H: Hookload
ST: Surface torque
RS: Rotary speed
Fi: Flow in
Fo: Flow out
Wp: Wellhead pressure
AID: Automatic interaction detection
AI: Artificial intelligence
RPM: Revolutions per minute
UCS: Uniaxial compressive strength
PV: Plastic viscosity
MW: Mud weight
YP: Yield point
ELM: Extreme learning machine
THAID: THeta Automatic Interaction Detection
GRG: Generalized reduced gradient
LSSVM: Least Square Support Vector Machine
PDC: Polycrystalline diamond compact
SVR: Support vector regression
CSVR-ICA: Committee support vector regression based on imperialist competitive algorithm
CIT: Computational intelligence techniques
LS-SVR: Least-square support vector regression
ANFIS: Adaptive neuro-fuzzy inference system
SVM: Support vector machine

## References

[1] Bahari MH, Bahari A, Moradi H (2011). Intelligent drilling rate predictor. Int. J. Innov. Comput. Inf. Control..

[2] Hadi HA, Engineering P (2015). Correlation of penetration rate with drilling parameters for an Iraqi field using mud logging data. Iraqi J. Chem. Petrol. Eng..

[3] Kaiser MJ (2007). Technology: a survey of drilling cost and complexity estimation models. Int. J. Petrol. Sci. Technol..

[4] Barbosa LFF, Nascimento A, Mathias MH, de Carvalho Jr JA (2019). Machine learning methods applied to drilling rate of penetration prediction and optimization: a review. J. Petrol. Sci. Eng..

[5] Metropolis N, Rosenbluth AW, Rosenbluth MN, Teller AH, Teller E (1953). Equation of state calculations by fast computing machines. J. Chem. Phys..

[6] Soares C, Gray K (2019). Real-time predictive capabilities of analytical and machine learning rate of penetration (ROP) models. J. Petrol. Sci. Eng..

[7] Akgun F (2007). Drilling rate at the technical limit. J. Petrol. Sci. Technol..

[8] Bataee M, Irawan S, Kamyab M (2014). Artificial neural network model for prediction of drilling rate of penetration and optimization of parameters. J. Jpn. Petrol. Inst..

[9] Paone, J., Madson, D. *Drillability Studies: Impregnated Diamond Bits.* Department of the Interior, Bureau of Mines (1966).

[10] Khosravanian R, Sabah M, Wood DA, Shahryari A (2016). Weight on drill bit prediction models: Sugeno-type and Mamdani-type fuzzy inference systems compared. J. Nat. Gas Sci. Eng..

[11] Paone, J., Bruce, W. E., Virciglio, P. R. *Drillability Studies: Statistical Regression Analysis of Diamond Drilling.* US Dept. of the Interior, Bureau of Mines (1966).

[12] Ayoub M, Shien G, Diab D, Ahmed Q (2017). Modeling of drilling rate of penetration using adaptive neuro-fuzzy inference system. Int J Appl Eng Res.

[13] Ersoy, A., Waller, M. Prediction of drill-bit performance using multi-variable linear regression analysis. In *International Journal of Rock Mechanics and Mining Sciences and Geomechanics Abstracts.* **6**, 279A (1995).

[14] Mendes, J. R. P., Fonseca, T. C., Serapião, A. Applying a genetic neuro-model reference adaptive controller in drilling optimization. 29–36 (2007).

[15] Mitchell, R., Miska, S. *Fundamentals of Drilling Engineering*; Society of Petroleum Engineers, Inc.: Richardson, TX, USA, 2011; Chapter 4. Google Scholar.

[16] Maurer W (1962). The, "perfect-cleaning" theory of rotary drilling. J. Petrol. Technol..

[17] Bingham G. A new approach to interpreting rock drillability. *Tech. Manual Reprint Oil Gas J.*, **93**, 1965 (1965).

[18] Bourgoyne, Jr. A. T., Millheim, K. K., Chenevert, M. E., Young, Jr F.S. Applied drilling engineering. (1991).

[19] Bourgoyne AT, Young F (1974). A multiple regression approach to optimal drilling and abnormal pressure detection. Soc. Petrol. Eng. J..

[20] Eren T, Ozbayoglu ME (2010). Real time optimization of drilling parameters during drilling operations.

[21] Hareland, G., Rampersad, P. Drag-bit model including wear. In *SPE Latin America/Caribbean Petroleum Engineering Conference.* Society of Petroleum Engineers; 1994.

[22] Soares C, Daigle H, Gray K (2016). Evaluation of PDC bit ROP models and the effect of rock strength on model coefficients. J. Nat. Gas Sci. Eng..

[23] Motahhari HR, Hareland G, James J (2010). Improved drilling efficiency technique using integrated PDM and PDC bit parameters. J. Can. Pet. Technol..

[24] Deng, Y., Chen, M., Jin, Y., Zhang, Y., Zou, D., Lu, Y., et al. Theoretical and experimental study on the penetration rate for roller cone bits based on the rock dynamic strength and drilling parameters. **36**, 117–123 (2016).

[25] Al-AbdulJabbar, A., Elkatatny, S., Mahmoud, M., Abdelgawad, K., Al-Majed, A. A robust rate of penetration model for carbonate formation. *J. Energy Resour. Technol.* **141**(4) (2019).

[26] Elkatatny, S. New approach to optimize the rate of penetration using artificial neural network. 1–8 (2017).

[27] Warren, T. J. S. D. E. Penetration rate performance of roller cone bits. **2**(01):9–18 (1987).

[28] Hareland, G., Hoberock, L. Use of drilling parameters to predict in-situ stress bounds. In *SPE/IADC Drilling Conference.* Society of Petroleum Engineers (1993).

[29] Hareland, G., Wu, A., Rashidi, B. A drilling rate model for roller cone bits and its application. In *International Oil and Gas Conference and Exhibition in China.* Society of Petroleum Engineers (2010).

[30] Hareland G, Wu A, Rashidi B, James J. A new drilling rate model for tricone bits and its application to predict rock compressive strength. In *44th US Rock Mechanics Symposium and 5th US-Canada Rock Mechanics Symposium.* American Rock Mechanics Association (2010).

[31] Eckel JR. Microbit studies of the effect of fluid properties and hydraulics on drilling rate, ii. In *Fall Meeting of the Society of Petroleum Engineers of AIME.* Society of Petroleum Engineers; 1968.

[32] Paiaman, A. M., Al-Askari, M., Salmani, B., Alanazi, B. D., Masihi, M. J. N. Effect of drilling fluid properties on rate of Penetration. **60**(3), 129–34 (2009).

[33] Moraveji, M. K., Naderi, M. Drilling rate of penetration prediction and optimization using response surface methodology and bat algorithm. **31**, 829–41 (2016).

[34] Arabjamaloei R, Shadizadeh S (2011). Modeling and optimizing rate of penetration using intelligent systems in an Iranian southern oil field (Ahwaz oil field). Pet. Sci. Technol..

[35] Amar, K., Ibrahim, A. Rate of penetration prediction and optimization using advances in artificial neural networks, a comparative study. In *4th International Joint Conference on Computational Intelligence,* 647–52 (2012).

[36] Hegde C, Daigle H, Millwater H, Gray K (2017). Analysis of rate of penetration (ROP) prediction in drilling using physics-based and data-driven models. J. Petrol. Sci. Eng..

[37] Bilgesu H, Tetrick L, Altmis U, Mohaghegh S, Ameri S (1997). A new approach for the prediction of rate of penetration (ROP) values.

[38] AlArfaj, I., Khoukhi, A., Eren, T. Application of advanced computational intelligence to rate of penetration prediction. In *Computer Modeling and Simulation (EMS), 2012 Sixth UKSim/AMSS European Symposium on.* IEEE; 33–38 (2012).

[39] Ansari HR, Hosseini MJS, Amirpour M (2017). Drilling rate of penetration prediction through committee support vector regression based on imperialist competitive algorithm. Carbonates Evaporites.

[40] Ashrafi SB, Anemangely M, Sabah M, Ameri MJ (2019). Application of hybrid artificial neural networks for predicting rate of penetration (ROP): a case study from Marun oil field. J. Petrol. Sci. Eng..

[41] Diaz MB, Kim KY, Shin H-S, Zhuang L (2019). Predicting rate of penetration during drilling of deep geothermal well in Korea using artificial neural networks and real-time data collection. J. Nat. Gas Sci. Eng..

[42] Gan C, Cao W-H, Wu M, Chen X, Hu Y-L, Liu K-Z (2019). Prediction of drilling rate of penetration (ROP) using hybrid support vector regression: a case study on the Shennongjia area, Central China. J. Petrol. Sci. Eng..

[43] Mehrad, M., Bajolvand, M., Ramezanzadeh, A., Neycharan, J. G. Developing a new rigorous drilling rate prediction model using a machine learning technique. *J. Petrol. Sci. Eng.* 107338 (2020).

[44] Gill, P. E., Murray, W., Wright, M. H. *Practical Optimization*. Academic Press, New York (1981).

[45] Ameli F, Hemmati-Sarapardeh A, Dabir B, Mohammadi AH (2016). Determination of asphaltene precipitation conditions during natural depletion of oil reservoirs: a robust compositional approach. Fluid Phase Equilib..

[46] Wilde, D. J., Beightler, C. S. *Foundations of Optimization* (1967).

[47] Sharma R, Glemmestad B (2013). On generalized reduced gradient method with multi-start and self-optimizing control structure for gas lift allocation optimization. J. Process Control.

[48] David CY, Fagan JE, Foote B, Aly AA (1986). An optimal load flow study by the generalized reduced gradient approach. Electric Power Syst. Res..

[49] Abadie, J. Generalization of the Wolfe reduced gradient method to the case of nonlinear constraints. *Optimization* 37–47 (1969).

[50] Morgan JN, Sonquist JA (1963). Problems in the analysis of survey data, and a proposal. J. Am. Stat. Assoc..

[51] Messenger R, Mandell L (1972). A modal search technique for predictive nominal scale multivariate analysis. J. Am. Stat. Assoc..

[52] Song Y-Y, Ying L (2015). Decision tree methods: applications for classification and prediction. Shanghai Arch. Psychiatry.

[53] Patel N, Upadhyay S. Study of various decision tree pruning methods with their empirical comparison in WEKA. *Int. J. Comput. Appl.* **60**(12) (2012).

[54] Ameli, F., Hemmati-Sarapardeh, A., Schaffie, M., Husein, M. M., Shamshirband, S. J. F. Modeling interfacial tension in N 2/n-alkane systems using corresponding state theory: application to gas injection processes. **222, **779–791 (2018).

[55] Hemmati-Sarapardeh, A., Varamesh, A., Husein, M. M., Karan, K. J. R., Reviews, S. E. On the evaluation of the viscosity of nanofluid systems: modeling and data assessment. **81**, 313–329 (2018).

[56] Karkevandi-Talkhooncheh, A., Rostami, A., Hemmati-Sarapardeh, A., Ahmadi, M., Husein, M. M., Dabir, B. J. F. Modeling minimum miscibility pressure during pure and impure CO_2_ flooding using hybrid of radial basis function neural network and evolutionary techniques. **220**, 270–282 (2018).

[57] Varamesh, A., Hemmati-Sarapardeh, A., Dabir, B., Mohammadi, A. H. Development of robust generalized models for estimating the normal boiling points of pure chemical compounds. **242**, 59–69 (2017).

[58] Rostami, A., Hemmati-Sarapardeh, A., Shamshirband, S. J. F. Rigorous prognostication of natural gas viscosity: smart modeling and comparative study. **222**, 766–778 (2018).

[59] Suykens JA, Vandewalle J (1999). Least squares support vector machine classifiers. Neural Process. Lett..

[60] Hagan MT, Menhaj MB (1994). Training feedforward networks with the Marquardt algorithm. IEEE Trans. Neural Networks.

[61] Yue, Z., Songzheng, Z., Tianshi, L. Bayesian regularization BP Neural Network model for predicting oil-gas drilling cost. In *2011 International Conference on Business Management and Electronic Information.* 2. IEEE; 483–487 (2011).

[62] Schapire RE (1990). The strength of weak learnability. Mach. Learn..

[63] Hastie T, Tibshirani R, Friedman J (2009). The Elements of Statistical Learning.

[64] Friedman JH (2002). Stochastic gradient boosting. Comput. Stat. Data Anal..

[65] Rostami, A., Baghban, A., Mohammadi, A. H., Hemmati-Sarapardeh, A., Habibzadeh, S. J. F. Rigorous prognostication of permeability of heterogeneous carbonate oil reservoirs: smart modeling and correlation development. **236**, 110–123 (2019).

[66] Tohidi-Hosseini S-M, Hajirezaie S, Hashemi-Doulatabadi M, Hemmati-Sarapardeh A, Mohammadi AH (2016). Toward prediction of petroleum reservoir fluids properties: a rigorous model for estimation of solution gas-oil ratio. J. Nat. Gas Sci. Eng..

[67] Leroy, A. M., Rousseeuw, P. J. Robust regression and outlier detection. rrod (1987).

[68] Hemmati-Sarapardeh A, Ameli F, Dabir B, Ahmadi M, Mohammadi AH (2016). On the evaluation of asphaltene precipitation titration data: Modeling and data assessment. Fluid Phase Equilib..

[69] Goodall, C. R. 13 Computation using the QR decomposition (1993).

[70] Gramatica P (2007). Principles of QSAR models validation: internal and external. QSAR Comb. Sci..

[71] Mohammadi AH, Eslamimanesh A, Gharagheizi F, Richon D (2012). A novel method for evaluation of asphaltene precipitation titration data. Chem. Eng. Sci..

[72] Mehrjoo, H., Riazi, M., Amar, M. N., Hemmati-Sarapardeh, A. Modeling interfacial tension of methane-brine systems at high pressure and high salinity conditions. *J. Taiwan Inst. Chem. Eng.* (2020).

